# Utilization of Filgrastim and Infliximab Biosimilar Products in Medicare Part D, 2015-2019

**DOI:** 10.1001/jamanetworkopen.2022.1117

**Published:** 2022-03-07

**Authors:** S. M. Qasim Hussaini, Arjun Gupta, Kelly E. Anderson, Jeromie M. Ballreich, Lauren Hersch Nicholas, G. Caleb Alexander

**Affiliations:** 1 Sidney Kimmel Comprehensive Cancer Center, Johns Hopkins Hospital, Baltimore, Maryland; 2 Masonic Cancer Center, University of Minnesota, Minneapolis; 3 Skaggs School of Pharmacy and Pharmaceutical Sciences, University of Colorado Anschutz Medical Campus, Aurora; 4 Department of Health Policy & Management, Johns Hopkins Bloomberg School of Public Health, Baltimore, Maryland; 5 Center for Drug Safety and Effectiveness, Johns Hopkins Bloomberg School of Public Health, Baltimore, Maryland; 6 Department of Health Systems, Management & Policy, University of Colorado Anschutz Medical Campus, Aurora; 7 Department of Epidemiology, Johns Hopkins Bloomberg School of Public Health, Baltimore, Maryland; 8 Division of General Internal Medicine, Johns Hopkins Medicine, Baltimore, Maryland

## Abstract

This cross-sectional study examines utilization trends for filgrastim and infliximab products and their biosimilars to understand whether biosimilars are associated with reduced spending in Medicare Part D.

## Introduction

The Biologics Price Competition and Innovation Act of 2009 created an approval pathway for biological products that are biosimilar or interchangeable with a US Food and Drug Administration–licensed originator biologic in the US. Filgrastim and infliximab were the first products with approved biosimilars. Filgrastim is used predominantly in treatment for cancer (eg, leukemia and solid organ cancer), and infliximab is used for autoimmune conditions (eg, ankylosing spondylitis and rheumatoid arthritis). While the majority of their claims are covered under Medicare Part B (primary outpatient benefit covering doctor visits) with administration in physician practices and outpatient departments, a portion are also covered under Medicare Part D (prescription drug benefit). With ongoing deliberations on drug pricing legislation that would enable government price negotiation, we sought to understand whether biosimilars are associated with reduced spending in Part D, where insurers and pharmacy benefit managers are able to negotiate drug prices.

## Methods

This cross-sectional study evaluated utilization trends for filgrastim products, including filgrastim (Neupogen, originator), tbo-filgrastim (Granix), and filgrastim-sndz (Zarxio), and infliximab products, including infliximab (Remicade, originator), infliximab-dyyb (Inflectra), and infliximab-abda (Renflexis), using the 2015 to 2019 Medicare Part D Drug Spending Dashboard. Because the data source is publicly available and the data are anonymous, the study was exempt from institutional review board approval and the need for informed consent, in accordance with 45 CFR §46. This report follows the Strengthening the Reporting of Observational Studies in Epidemiology (STROBE) reporting guideline.

We present inflation-adjusted annual spending (total, per beneficiary receiving drug in calendar year, per dosage unit), and total claims, for all filgrastim and infliximab formulations approved by 2017. Analyses were performed in Excel software version 16.56 (Microsoft Corp).

## Results

From 2015 to 2019, utilization of filgrastim and infliximab billed through Medicare Part D increased (Figure). For filgrastim, total claims increased by 15% from 27 026 to 31 156 claims; for infliximab, these increased by 41%, from 11 282 to 15 986 claims. Spending per beneficiary and dosage unit are noted in the Table. Between 2015 and 2019, originator filgrastim had a 5.3% ($435.40) reduction, and originator infliximab had a 37.5% ($9438.80) increase in spending per beneficiary.

**Figure.**
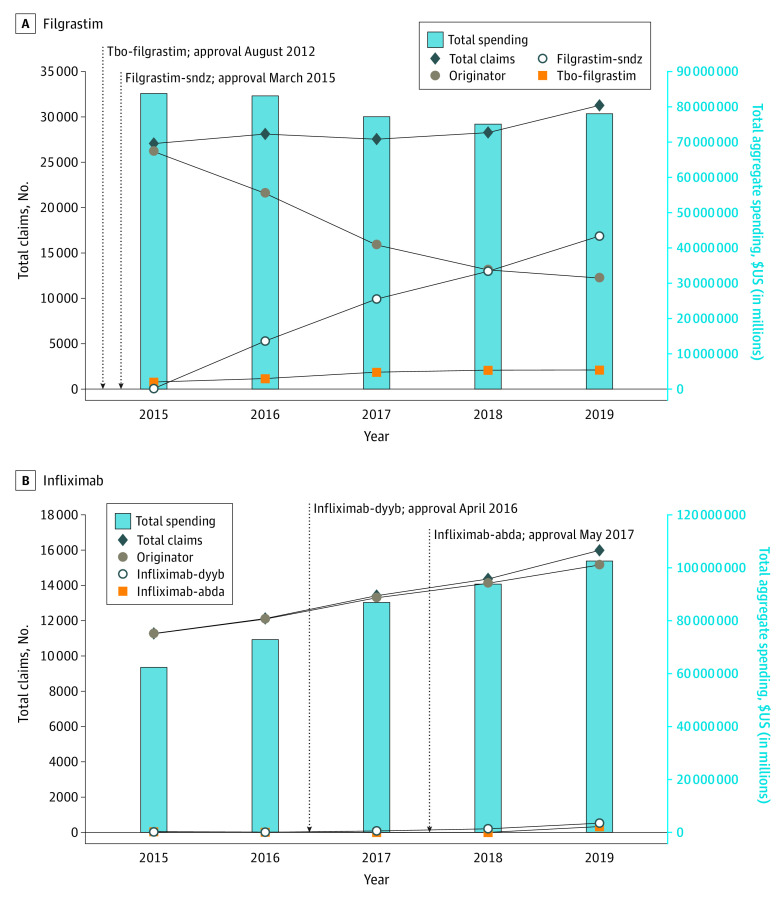
Time Trends in the Utilization of Filgrastim and Infliximab Products in Medicare Part D, 2015-2019 Graphs show data for filgrastim (A) and infliximab (B). This analysis used the 2015-2019 Medicare Part D Drug Spending Dashboard. Inset lines (dotted) note approval of biosimilars for each biologic. Filgrastim (Neupogen, originator) was originally approved in February 1991, tbo-filgrastim (Granix) was originally approved in August 2012, and filgrastim-sndz (Zarxio) was originally approved in March 2015. Infliximab (Remicade, originator) was originally approved in August 1998, infliximab-dyyb (Inflectra) was originally approved in April 2016, and infliximab-abda (Renflexis) was originally approved in April 2017. US dollar amounts are shown (inflation adjusted to 2019).

**Table.** Total Annual Spending on Filgrastim and Infliximab Products, 2015-2019^a^

Drug and year	Annual spending in millions, $US (% of total spending)			Spending per beneficiary, $US			Spending per dosage unit, $US		
	Originator	Filgrastim-sndz	Tbo-filgrastim	Originator	Filgrastim-sndz	Tbo-filgrastim	Originator	Filgrastim-sndz	Tbo-filgrastim
Filgrastim									
2015	82.1 (97.8)	0.08 (0.1)	1.8 (2.1)	8202.9	4310.2	5124.3	619.2	568.6	560.5
2016	66.8 (80.3)	13.1 (15.7)	3.3 (3.9)	8298.7	6528.3	5812.2	597.9	603.1	566.1
2017	47.5 (61.4)	24.8 (32.0)	4.9 (6.4)	8285.6	6583.8	5888.1	605.2	590.3	534.4
2018	38.3 (51.0)	31.4 (41.8)	5.4 (7.2)	8204.3	6551.2	6415.8	615.4	572.1	570.0
2019	34.1 (43.6)	39.0 (49.9)	4.9 (6.3)	7767.5	6612.2	5786.7	617.5	537.2	557.1
Infliximab	Originator	Infliximab-dyyb	Infliximab-abda	Originator	Infliximab-dyyb	Infliximab-abda	Originator	Infliximab-dyyb	Infliximab-abda
2015	62.3 (100)	NA	NA	25 167	NA	NA	864.5	NA	NA
2016	72.5 (100)	NA	NA	28 282.1	NA	NA	865.2	NA	NA
2017	86.7 (99.6)	0.31 (0.36)	NA	31 474.6	15 856.4	NA	1086.7	1010.4	NA
2018	92.5 (98.8)	1.1 (1.2)	0.44 (0.0)	32 669.0	19 789.1	NA	1094.1	984.6	792.6
2019	98.7 (96.2)	2.7 (2.5)	1.2 (1.1)	34 605.8	19 696.4	14 657.0	1045.9	891.9	778.3

Abbreviation: NA, not applicable.

^a^ Trends in annual spending, spending per beneficiary, and spending per dosage unit are shown from 2015-2019. This analysis used the 2015-2019 Medicare Part D Drug Spending Dashboard data for originator filgrastim and infliximab and their approved biosimilars. US dollar amounts, inflation adjusted to 2019, are shown.

The share of total annual spending on filgrastim biosimilars increased from $1.8 million (2% in 2015) to $44 million (56% in 2019), eclipsing the originator biologic in 2018. For infliximab, from 2017 (the year after the first biosimilar approval) to 2019, the biosimilar share increased from less than 1% to 3.6%. Total annual Part D spending on filgrastim decreased by only 7% from 2015 to 2019 (from $84 million to $78 million) (Figure), while total annual Part D spending on infliximab increased by 17% (from $87 million to $102 million) despite biosimilar competition.

## Discussion

Despite substantial uptake of filgrastim biosimilars in Medicare Part D, uptake of infliximab biosimilars remained low between 2017 and 2019. These differences may represent less frequent dosing of infliximab and varied perceptions of biosimilar efficacy across different clinician specialties.^1^ Compared with biosimilar uptake in Part B,^2,3^ lower uptake in Part D may reflect preferred formulary placement of originator biologics for Part D plans.^4^

In this cross-sectional study, we report increasing utilization for both filgrastim and infliximab products. Despite this, lower transaction prices resulted in lower total annual spending for filgrastim products. This is different from infliximab, where we observed marked increase in total spending. While conventional economic wisdom would suggest that biosimilar competition would reduce prices and spending per beneficiary, the complexity of the US pharmaceutical market, with proprietary discounts and rebates, may hinder market competition.^5^ Our analyses are limited by lack of data on rebates or discounts associated with formulary placement and prescribing patterns.

For policy makers, the lack of observed price competition from biosimilars should be concerning. As these are specialty drugs, most Part D plans would set cost sharing levels equal to a percentage of drug price, thereby preventing beneficiaries from reaping the full benefits of biosimilar competition. Redesigning the Part D benefit to include more favorable formulary placement or reduce prior authorization requirements for biosimilars could increase use.^4^

Finally, payment for filgrastim and infliximab predominantly occurs through Part B. A smaller volume of claims in Part D means that insurers may not have sufficient market power to leverage purchasing power. Thus, Congressional legislation should ensure purchasers have real power when negotiating with drug manufacturers, whether through representing a high-purchasing volume or through price limits such as internal or external reference pricing.^6^
